# Using capnography to differentiate airway from gastrointestinal placement of nasogastric tubes: a prospective multicentre diagnostic accuracy study

**DOI:** 10.1186/s13054-026-06246-9

**Published:** 2026-08-14

**Authors:** Janita Pak Chun Chau, Danny Wah Kun Tong, Suzanne Hoi Shan Lo, Savina Yee Man Sze, Peter Chi Keung Lai, Josephine Yuen Man Chung, Molly Lai Mei Kwok, Harris Kai Cheong Lam, Ying Hei Fan, Wai Tong Chien, Philip Wing Ping Au, Kai Chow Choi

**Affiliations:** 1 The Nethersole School of Nursing, Faculty of Medicine, The Chinese University of Hong Kong, Shatin, N.T., Hong Kong SAR China; 2 Nursing Services Department, Hospital Authority Head Office, Hospital Authority, 147B Argyle Street, Kowloon, Hong Kong SAR China; 3 Department of Medicine and Geriatrics, Shatin Hospital, Hospital Authority, 33 A Kung Kok Street, Ma On Shan, Shatin, N.T., Hong Kong SAR China; 4 Adult Intensive Care Unit, Queen Mary Hospital, Hospital Authority, 102 Pok Fu Lam Road, Pok Fu Lam, Hong Kong Island, Hong Kong SAR China; 5 Alice Ho Miu Ling Nethersole Hospital & Tai Po Hospital, Hospital Authority, 9 Chuen On Road, Tai Po, N.T., Hong Kong SAR China; 6 Accident and Emergency Department, Prince of Wales Hospital, Hospital Authority, 30-32 Ngan Shing Street, Shatin, N.T., Hong Kong SAR China

**Keywords:** Capnography, Cost analysis, Diagnostic accuracy, End-tidal carbon dioxide, Gastrointestinal intubation, Inadvertent airway placement, Nasogastric tube, Radiology, Tube misplacement

## Abstract

**Background:**

Patient safety incident reports and case reports have repeatedly highlighted factors contributing to inadvertent airway placement of nasogastric tubes, including misleading pH readings, insufficiently documented placement checks, and misinterpretation of radiographs. The study aimed to: (i) evaluate the diagnostic performance of capnography compared with radiography for detecting inadvertent airway placement of nasogastric tubes; and (ii) estimate the average cost incurred when radiography is used alone versus when capnography is used in conjunction with radiography.

**Methods:**

A multicentre, prospective, observational diagnostic study was conducted. The diagnostic accuracy of capnography was examined using three end-tidal carbon dioxide thresholds (> 5 mmHg, > 10 mmHg, > 15 mmHg), with radiographic confirmation as the reference standard. For each threshold, true positives, false negatives, true negatives, and false positives were determined. Cost savings per patient were estimated using a bias-corrected and accelerated bootstrapping method with 20,000 replications.

**Results:**

A total of 508 participants were recruited from 39 study sites across 21 hospitals, including medical or geriatric wards, intensive care units, and emergency departments. Most participants (*n* = 485; 95.5%) had end-tidal carbon dioxide readings ≤ 5 mmHg. Radiography confirmed correct gastric placement in 452 cases (89.0%), with one case (0.2%) of inadvertent airway placement. Estimated cost savings per patient, were HK$210.3, HK$214.5, and HK$217.5 for the three respective end-tidal carbon dioxide thresholds, based on a hypothetical strategy in which radiographic confirmation was performed only when the end-tidal carbon dioxide value exceeded a predefined threshold, compared with a radiography-only strategy in which all patients underwent radiographic confirmation of nasogastric tube placement.

**Conclusions:**

Given the favourable outcomes and the simplicity of capnography measurement, capnography can be considered a first-line tool for excluding airway placement of nasogastric tubes, used alongside pH testing of aspirates.

**Trial registration:**

ClinicalTrials.gov, Number NCT05817864 (Registration date: 18 April 2023).

**Supplementary Information:**

The online version contains supplementary material available at https://doi.org/10.1186/s13054-026-06246-9.

## Background

Inadvertent insertion of a nasogastric tube (NGT) into the airway is a serious iatrogenic complication that can result in pulmonary aspiration and death [1]. Although radiographic confirmation is regarded as the gold standard for verifying tube placement, it is not always immediately available. Bedside pH testing of aspirated fluid is commonly used as an alternative, but a lack of aspirates often limits its utility. Other methods used to verify NGT placement include assessment of tube aspirates for bilirubin, pepsin and trypsin levels; air auscultation (the whoosh test); the use of a spring-gauge pressure manometer; magnetic detection; sonography; and visual inspection of aspirates. However, these methods have several limitations, including difficulties in obtaining aspirates, the need for specialised equipment and technical expertise, and reliance on subjective interpretation. Consequently, their diagnostic accuracy has varied across studies, limiting their routine use in clinical practice [2, 3].

Patient safety incident reports have highlighted contributing factors in airway misplacement events, including apparently misleading pH results, placement checks not clearly described, and misinterpretation of X-rays [4]. Previous systematic reviews have reported that end-tidal carbon dioxide (ETCO₂) detection using capnography or colorimetric CO_2_ detection may offer diagnostic accuracy comparable to radiography in distinguishing airway from gastrointestinal (GI) placement of NGTs in critically ill adults [5, 6]. However, the paucity of studies undertaken in general wards and emergency care settings limits the ability to derive robust estimates of diagnostic accuracy for these patient populations. While earlier trials reported cost savings when using colorimetric CO₂ detectors and pH paper compared with radiography [7], evidence regarding cost savings when using radiography alone compared to when capnography is added remains limited. This study, therefore, aimed to evaluate the diagnostic performance of capnography compared with radiography in detecting inadvertent airway placement of NGTs, and to determine the average cost incurred by participants when radiography is used alone versus when used in conjunction with capnography. The objective is to generate robust evidence to inform clinical decision-making and support strategies that reduce the risks associated with airway misplacement of NGTs.

## Methods

All authors had access to the study data and reviewed and approved the final manuscript.

### Study design

A multicentre, prospective, observational diagnostic study was conducted. The study was registered with ClinicalTrials.gov, and its reporting adhered to the Standards for Reporting Diagnostic Accuracy (STARD) guidelines [8].

### Participants

We recruited patients who were aged ≥ 18 years; admitted to general medical or geriatric wards in acute, subacute, convalescent, or extended-care hospitals, admitted to intensive care units (ICUs), or attended emergency departments (EDs) in acute hospitals; and required NGT insertion for assessment and monitoring, nutritional support, drainage, or medication administration. Patients were excluded if they were undergoing life-saving interventions at the time of recruitment (e.g., cardiopulmonary resuscitation); exhibited signs of respiratory distress during insertion (e.g., vigorous coughing, choking, or dyspnoea) [9]; or were receiving invasive mechanical ventilation with a positive end-expiratory pressure (PEEP) ≥ 10 cmH₂O, as brief disconnection of the ventilator circuit during capnography device function checking may result in loss of PEEP and alveolar derecruitment, potentially placing patients with higher PEEP requirements at increased risk. As noted by Gallagher [10], interruption of ventilation in patients requiring PEEP ≥ 10 cmH₂O should generally be avoided, as restoration of functional residual capacity and arterial oxygenation following derecruitment may be prolonged.

Anticipating sensitivity and specificity values of capnography in detecting inadvertent airway placement of NGTs exceeding 90% [6], a sample size of at least 500 participants was required to estimate these parameters with a margin of error of at most ± 3% (significance level: 0.05) using PASS 16 (NCSS, Kaysville, USA).

### Test methods

**Fig. 1:**
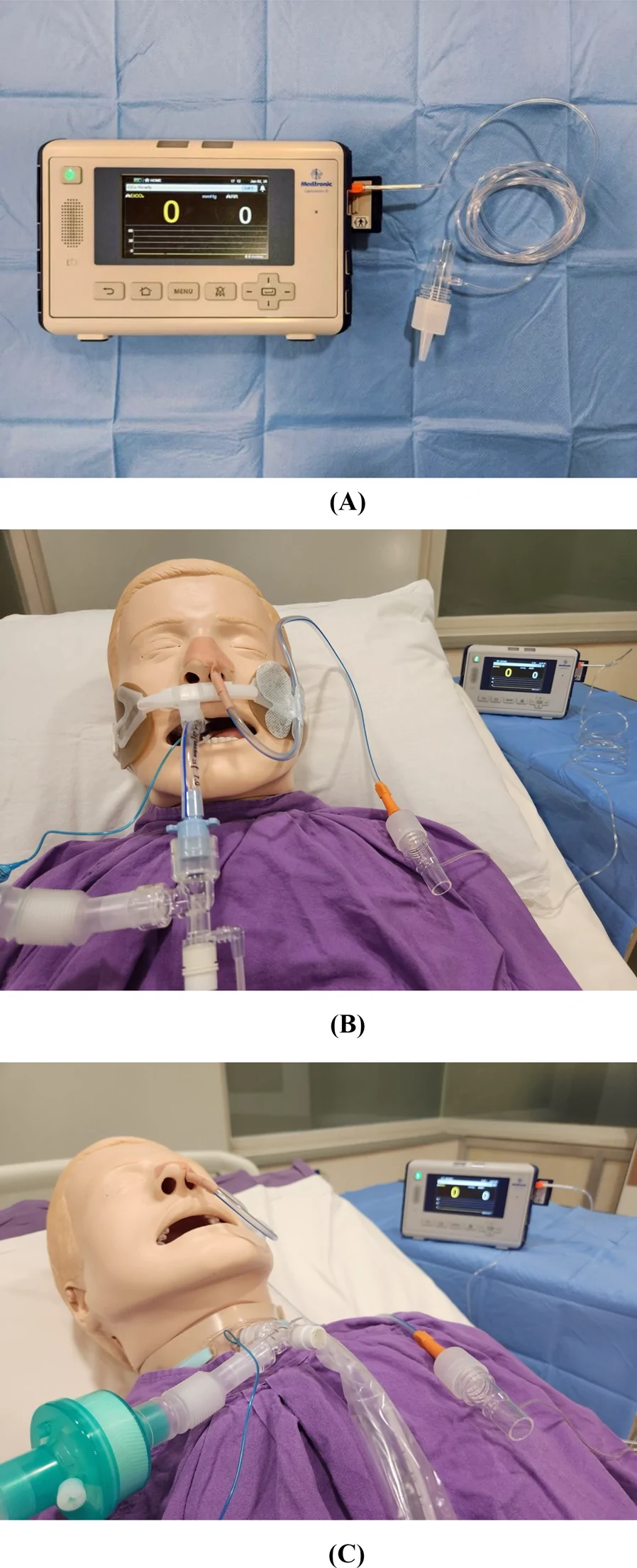
**A**. Connection of a sidestream capnography device to a nasogastric tube using a disposable CO₂ filter line and adaptor. **B**. Connection of a sidestream capnography device to a nasogastric tube in a manikin fitted with an endotracheal tube and attached to a mechanical ventilator and closed suction system. **C**. Connection of a sidestream capnography device to a nasogastric tube in a manikin fitted with a tracheostomy tube and connected to a mechanical ventilator and closed suction system

Capnography, which measures ETCO₂, the percentage concentration or partial pressure of CO_2_ at the end of exhalation, served as the index test. It was performed by connecting the distal end of the NGT to the sensor of a bedside sidestream capnography device (Medtronic Capnostream 35 Portable Respiratory Monitor) via a CO₂ filter line and adapter (Fig. 1A and C). The waveform capnography device offers continuous waveform monitoring in addition to numerical ETCO₂ values, thereby delivering more comprehensive information [11]. After the NGT was connected, the ETCO₂ value was recorded after a minimum of six breaths or one minute, to reduce the influence of residual gastric CO₂ [12, 13].

In this study, airway placement of an NGT was defined as the presence of a normal CO₂ waveform (capnogram) together with a positive ETCO₂ reading. Previous studies have applied varying thresholds for defining a positive index test: Ryu et al. used an ETCO₂ threshold of > 5 mmHg to indicate airway placement [14], whereas Burns et al. defined lung or airway placement as ETCO₂ >15 mmHg [15]. Ceruti et al. reported a mean oesophageal ETCO₂ of 11 mmHg (SD 9.3) in mechanically ventilated patients [16]. To evaluate diagnostic accuracy across a range of clinically relevant thresholds, this study presents results using ETCO₂ thresholds of > 5 mmHg, > 10 mmHg, and > 15 mmHg.

Radiography (chest X-ray and/or abdominal X-rays) was used as the reference standard, as radiologic confirmation is recommended by eleven of fourteen international guidelines for distinguishing stomach from airway NGT placement [17]. Radiographic imaging was performed after completion of the index test and was interpreted by a physician or radiologist. Repeat radiography when necessary to verify tube position.

A standardised protocol detailing the procedures for ETCO₂ detection and data collection was developed to minimise erroneous results arising from improper operation of the bedside sidestream capnography device and to ensure research fidelity [18]. All designated nurses received investigator-led training and were required to pass both a quiz and a competency skill audit in capnography assessment.

Designated nurses at each study site screened participants according to the eligibility criteria. For participants requiring NGT insertion, the designated nurse set up the capnography device and verified its function by placing the sampling port at the nostrils of non-intubated patients, or at the artificial airway of intubated patients. Proper device function was confirmed by the presence of capnography waveform and an ETCO_2_ value.

Ward nurses performed NGT insertion, estimating the insertion depth using the nose–earlobe–xiphoid method and adhering to the Hospital Authority clinical practice guidelines. They flushed the tube with air to confirm patency and checked for any coiling of the NGT within the oral cavity. Afterwards, designated nurses independently performed the index test by observing the capnography waveform and recording the ETCO₂ value. Ward nurses then independently aspirated fluid (if obtainable) for pH measurement and documented the result.

To minimise bias, the designated nurses did not disclose the capnography results to the ward nurses. Subsequently, each patient underwent a chest X-ray and/or an abdominal X-ray, which was interpreted by a physician or radiologist to confirm correct NGT placement. Additional measures to mitigate potential bias included ensuring that index test results were not available to the assessors of the reference standard and that no clinical interventions occurred between the index test and the reference standard. All machine-generated reports containing continuous capnography waveforms and ETCO₂ values were also reviewed by the investigators to verify the accuracy of the nurses’ ETCO₂ documentation.

### Ethical considerations

This study involved human participants and adhered to the Declaration of Helsinki. It was approved by The Joint Chinese University of Hong Kong-New Territories East Cluster Clinical Research Ethics Committee (CREC Ref. No. 2022.073), Central Institutional Review Board (CIRB-2023-030-4), and Institutional Review Board of the University of Hong Kong / Hospital Authority Hong Kong West Cluster (HKU/HA HKW IRB Ref. No. UW 23–332). Participants provided informed consent before participating in the study.

### Statistical analysis

Descriptive statistics were used to summarise and present participants’ demographic and clinical characteristics. The diagnostic performance of capnography in identifying inadvertent airway placement of NGTs was evaluated using three ETCO₂ thresholds: (i) > 5 mmHg, (ii) > 10 mmHg, and (iii) > 15 mmHg, with radiographic findings serving as the reference standard. For each threshold, the numbers of true positives (NGT airway placements positive on both the index test and reference standard), false negatives (negative on the index test but positive on the reference standard), true negatives (negative on both index test and reference standard), and false positives (positive on the index test but negative on the reference standard) were determined. Based on these values, sensitivity, specificity, positive predictive value (PPV), and negative predictive value (NPV) were calculated using the following formulas:

$$\begin{aligned}\:\mathrm{S}\mathrm{e}\mathrm{n}\mathrm{s}\mathrm{i}\mathrm{t}\mathrm{i}\mathrm{v}\mathrm{i}\mathrm{t}\mathrm{y}&=\mathrm{T}\mathrm{r}\mathrm{u}\mathrm{e}\:\mathrm{P}\mathrm{o}\mathrm{s}\mathrm{i}\mathrm{t}\mathrm{i}\mathrm{v}\mathrm{e}/(\mathrm{T}\mathrm{r}\mathrm{u}\mathrm{e}\:\mathrm{P}\mathrm{o}\mathrm{s}\mathrm{i}\mathrm{t}\mathrm{i}\mathrm{v}\mathrm{e}\\&\quad\:+\mathrm{F}\mathrm{a}\mathrm{l}\mathrm{s}\mathrm{e}\:\mathrm{N}\mathrm{e}\mathrm{g}\mathrm{a}\mathrm{t}\mathrm{i}\mathrm{v}\mathrm{e})\times100{\%}\end{aligned}$$

$$\begin{aligned}\:\mathrm{S}\mathrm{p}\mathrm{e}\mathrm{c}\mathrm{i}\mathrm{f}\mathrm{i}\mathrm{c}\mathrm{i}\mathrm{t}\mathrm{y}&=\mathrm{T}\mathrm{r}\mathrm{u}\mathrm{e}\:\mathrm{N}\mathrm{e}\mathrm{g}\mathrm{a}\mathrm{t}\mathrm{i}\mathrm{v}\mathrm{e}/(\mathrm{T}\mathrm{r}\mathrm{u}\mathrm{e}\:\mathrm{N}\mathrm{e}\mathrm{g}\mathrm{a}\mathrm{t}\mathrm{i}\mathrm{v}\mathrm{e}\\&\quad\:+\mathrm{F}\mathrm{a}\mathrm{l}\mathrm{s}\mathrm{e}\:\mathrm{P}\mathrm{o}\mathrm{s}\mathrm{i}\mathrm{t}\mathrm{i}\mathrm{v}\mathrm{e})\times100{\%}\end{aligned}$$

$$\begin{aligned}\:\mathrm{P}\mathrm{P}\mathrm{V}&=\mathrm{T}\mathrm{r}\mathrm{u}\mathrm{e}\:\mathrm{P}\mathrm{o}\mathrm{s}\mathrm{i}\mathrm{t}\mathrm{i}\mathrm{v}\mathrm{e}/(\mathrm{T}\mathrm{r}\mathrm{u}\mathrm{e}\:\mathrm{P}\mathrm{o}\mathrm{s}\mathrm{i}\mathrm{t}\mathrm{i}\mathrm{v}\mathrm{e}\\&\quad+\mathrm{F}\mathrm{a}\mathrm{l}\mathrm{s}\mathrm{e}\:\mathrm{P}\mathrm{o}\mathrm{s}\mathrm{i}\mathrm{t}\mathrm{i}\mathrm{v}\mathrm{e})\times\:100{\%}\end{aligned}$$

$$\begin{aligned}\:\mathrm{N}\mathrm{P}\mathrm{V}&=\mathrm{T}\mathrm{r}\mathrm{u}\mathrm{e}\:\mathrm{N}\mathrm{e}\mathrm{g}\mathrm{a}\mathrm{t}\mathrm{i}\mathrm{v}\mathrm{e}/(\mathrm{T}\mathrm{r}\mathrm{u}\mathrm{e}\:\mathrm{N}\mathrm{e}\mathrm{g}\mathrm{a}\mathrm{t}\mathrm{i}\mathrm{v}\mathrm{e}\\&\quad+\mathrm{F}\mathrm{a}\mathrm{l}\mathrm{s}\mathrm{e}\:\mathrm{N}\mathrm{e}\mathrm{g}\mathrm{a}\mathrm{t}\mathrm{i}\mathrm{v}\mathrm{e})\times\:100{\%}\end{aligned}$$

Positive and negative likelihood ratios were also computed to further assess diagnostic performance. An index test with positive likelihood ratio over 10 and a negative likelihood ratio below 0.1 is considered to have strong evidence of diagnostic ability for determining and eliminating a condition [19].

A cost analysis was conducted to evaluate the total costs incurred by participants under a radiography-only strategy and under each of the three capnography threshold strategies for identifying inadvertent airway placement of NGTs. The cost evaluation was designed as a hypothetical comparison of alternative clinical verification strategies rather than a comparison of the actual procedures performed during the study.

Specifically, we compared (1) a radiography-only strategy, in which all patients would undergo radiographic confirmation of NGT placement, with (2) a combined capnography and radiography strategy, in which radiographic confirmation would only be performed when the ETCO_2_ value exceeded a predefined threshold and further confirmation was required. Under the combined strategy, patients with ETCO_2_ values below the threshold would not require radiography confirmation; therefore, the associated radiography costs were not included in the analysis.

As both radiography and capnography equipment are routinely available in clinical settings, their acquisition and maintenance costs were not included in the analysis. The average cost saved per patient by using each capnography index test compared with using radiography only was estimated together with a confidence interval based on a biased-corrected and accelerated bootstrapping method with 20,000 replications [20]. All cost data were expressed in Hong Kong dollars (HK$) (1 US$~=7.78 HK$) and valued based on non-subsidised costs on the study start date.

The average waiting time for X-ray confirmation of NGT location was documented from the completion of the index test to the confirmation of NGT location by radiography.

All statistical analyses were performed using MedCalc Statistical Software version 23.0 (MedCalc Software Ltd., Ostend, Belgium; https://www.medcalc.org) and IBM SPSS Statistics version 30.0 (IBM Corp., Armonk, NY).

## Results

### Participants

Between 2023 and 2025, 643 patients were assessed for initial eligibility and invited to participate (Table 1). A total of 135 were excluded due to: failure to obtain consent (*n* = 78), NGT replacement before the scheduled index test (*n* = 16), meeting exclusion criteria (*n* = 9), pH testing performed before the index test (*n* = 9), technical issues (*n* = 8), X-ray taken before the index test (*n* = 6), repeated recruitment (*n* = 4), and absence of X-ray confirmation (*n* = 3). One patient was withdrawn at family request (*n* = 1). A clinically complicated case involving a patient with a cervical spine injury who had undergone surgery was excluded because ward nurses encountered significant difficulty with NGT insertion and observed repeated tube coiling during the procedure.

**Table 1:** Demographic and clinical characteristics of the participants (*N* = 508)

Characteristics	Mean (SD) / *n* (%)
Age (years) ^†^	74.9 (15.5)
Gender	
Male	266 (52.4%)
Female	242 (47.6%)
Clinical Frailty Scale	
1 (Very fit)	9 (1.8%)
2 (Well)	17 (3.3%)
3 (Managing well)	72 (14.2%)
4 (Vulnerable/pre-frail)	41 (8.1%)
5 (Mildly frail)	20 (3.9%)
6 (Moderately frail)	60 (11.8%)
7 (Severely frail)	164 (32.3%)
8 (Very severely frail)	124 (24.4%)
9 (Terminally ill)	1 (0.2%)
Glasgow Coma Scale	
*Eye opening*	
Not testable	12 (2.4%)
1	46 (9.0%)
2	31 (6.1%)
3	60 (11.8%)
4	359 (70.7%)
*Verbal response*	
Not testable	134 (26.4%)
1	87 (17.1%)
2	108 (21.3%)
3	16 (3.1%)
4	61 (12.0%)
5	102 (20.1%)
*Best motor response*	
Not testable	15 (3.0%)
1	59 (11.6%)
2	16 (3.2%)
3	26 (5.1%)
4	63 (12.4%)
5	114 (22.4%)
6	215 (42.3%)
*Combined score*	
Not testable	134 (26.4%)
< 9	73 (14.4%)
≥ 9	301 (59.2%)
Admission department/unit	
Medical or geriatric ward	120 (23.6%)
ED	218 (42.9%)
ICU including CTSD	170 (33.5%)
* Among those who were admitted to ED (n = 218)* Transferred from nursing homes/residential care facilities	135 (61.9%)
* Among those who were in ICU/CTSD (n = 170)*	
Unassisted spontaneous breathing	57 (33.5%)
Non-invasive ventilation support	9 (5.3%)
Presence of artificial airway (ETT)	97 (57.1%)
Presence of artificial airway (Tracheostomy)	18 (10.6%)
Type of X-ray performed	
CXR	495 (97.4%)
CXR + AXR	13 (2.6%)
NGT position on CXR (1st)	
Stomach	452 (89%)
Tracheobronchial tree	1 (0.2%)
Oesophagus	17 (3.3%)
Gastrointestinal tract (above diaphragm)	12 (2.4%)
Gastrointestinal tract (at diaphragm)	6 (1.2%)
At oesophageal-gastric junction	2 (0.4%)
Small bowel	2 (0.4%)
At midline	1 (0.2%)
Coiled in the nasopharynx	1 (0.2%)
Coiled in the mouth	4 (0.8%)
Coiled in the oropharynx	1 (0.2%)
Coiled in the oesophagus	6 (1.1%)
Coiled/kinked in the stomach	2 (0.4%)
Coiled (no location given)	1 (0.2%)
Waiting time for X-ray confirmation of NGT location	
From index test (with aspirate pH test) to confirmation of NGT location by radiography (Minutes) ^†^	162.7 (161.4)
From index test (without aspirate pH test) to confirmation of NGT location by radiography (Minutes) ^†^	122.5 (129.5)
Average waiting time from index test to confirmation of NGT location by radiography (Minutes) ^†^	148.6 (150.2)
Need for additional X-ray	
No	458 (90.2%)
Yes	50 (9.8%)
NGT position on chest x-ray (2nd)	
Stomach	37 (74.0%)
Oesophagus	6 (12.0%)
Small bowel	1 (2.0%)
Gastrointestinal tract (above diaphragm)	2 (4.0%)
Gastrointestinal tract (at diaphragm)	1 (2.0%)
At oesophageal-gastric junction	1 (2.0%)
Coiled in the mouth	1 (2.0%)
Coiled (no location given)	1 (2.0%)
Size of NGT	
8	1 (0.2%)
12	93 (18.3%)
14	348 (68.5%)
16	66 (13.0%)
Type of nasogastric tube	
PVC	218 (42.9%)
Polyurethane	104 (20.5%)
Silicone	180 (35.4%)
PVC with phthalate	2 (0.4%)
Latex	4 (0.8%)
Indication for NGT insertion	
Assessment & monitoring	36 (7.1%)
Medication	196 (38.6%)
Drainage	40 (7.9%)
Nutrition support	447 (88.0%)
Assessment & monitoring + medication	14 (2.8%)
Assessment & monitoring + medication + Drainage	3(0.6%)
Indications for nutritional support	
Dysphagia	250 (49.2%)
Infections	14 (2.8%)
Respiratory failure	69 (13.6%)
Stroke	99 (19.5%)
Advanced cognitive impairment	55 (10.8%)
Motor neurone disease	2 (0.4%)
Parkinson’s disease	9 (1.8%)
Pressure injury	4 (0.8%)
Postoperative	34 (6.7%)
Others (e.g., cancer, poor oral intake)	38 (7.5%)
Methods to test capnography device	
Inserting the adaptor in the nostril	198 (39.0%)
Placing the adaptor close to the nose	182 (35.8%)
Patient on mechanical ventilation: connecting the adaptor to the ventilator	102 (20.1%)
Patient with a tracheostomy: connecting the adaptor to the tracheostomy tube/placing the adaptor close to the tracheostomy stoma)	26 (5.1%)
ETCO_2_ (mmHg)	
≤ 5	485 (95.4%)
> 5–10	6 (1.2%)
> 10–15	4 (0.8%)
> 15	13 (2.6%)
Aspirate pH test	
Nil aspirate	241 (47.4%)
≤ 5.5	206 (40.6%)
> 5.5	61 (12.0%)
The location of the nasogastric tube where a pH sample could be taken	
Stomach (*n* = 452)	253 (56.0%)
Tracheobronchial tree (*n* = 1)	0 (0.0%)
Oesophagus (*n* = 17)	3 (17.6%)
Gastrointestinal tract (above diaphragm) (*n* = 12)	4 (33.3%)
Gastrointestinal tract (at diaphragm) (*n* = 6)	1 (16.7%)
At oesophageal-gastric junction (*n* = 2)	0 (0.0%)
At midline (*n* = 1)	1 (100.0%)
Small bowel (*n* = 2)	1 (50.0%)
Coiled in the nasopharynx (*n* = 1)	1 (100.0%)
Coiled in the mouth (*n* = 4)	2 (50.0%)
Coiled in the oropharynx (*n* = 1)	0 (0.0%)
Coiled in the oesophagus (*n* = 6)	0 (0.0%)
Kinked/Coiled in the stomach (*n* = 2)	1 (50.0%)
Coiled (no location given) (*n* = 1)	0 (0.0%)

Data marked with ^†^ are presented as mean (standard deviation), all others are presented as frequency (%)

AXR, abdominal X-ray; CTSD, Department of Cardiothoracic Surgery, CXR, chest X-ray, ED, emergency department; ETT, endotracheal tube; ICU, intensive care unit; NGT, nasogastric tube; SD, standard deviation; PVC, polyvinyl chloride

**Fig. 2:**
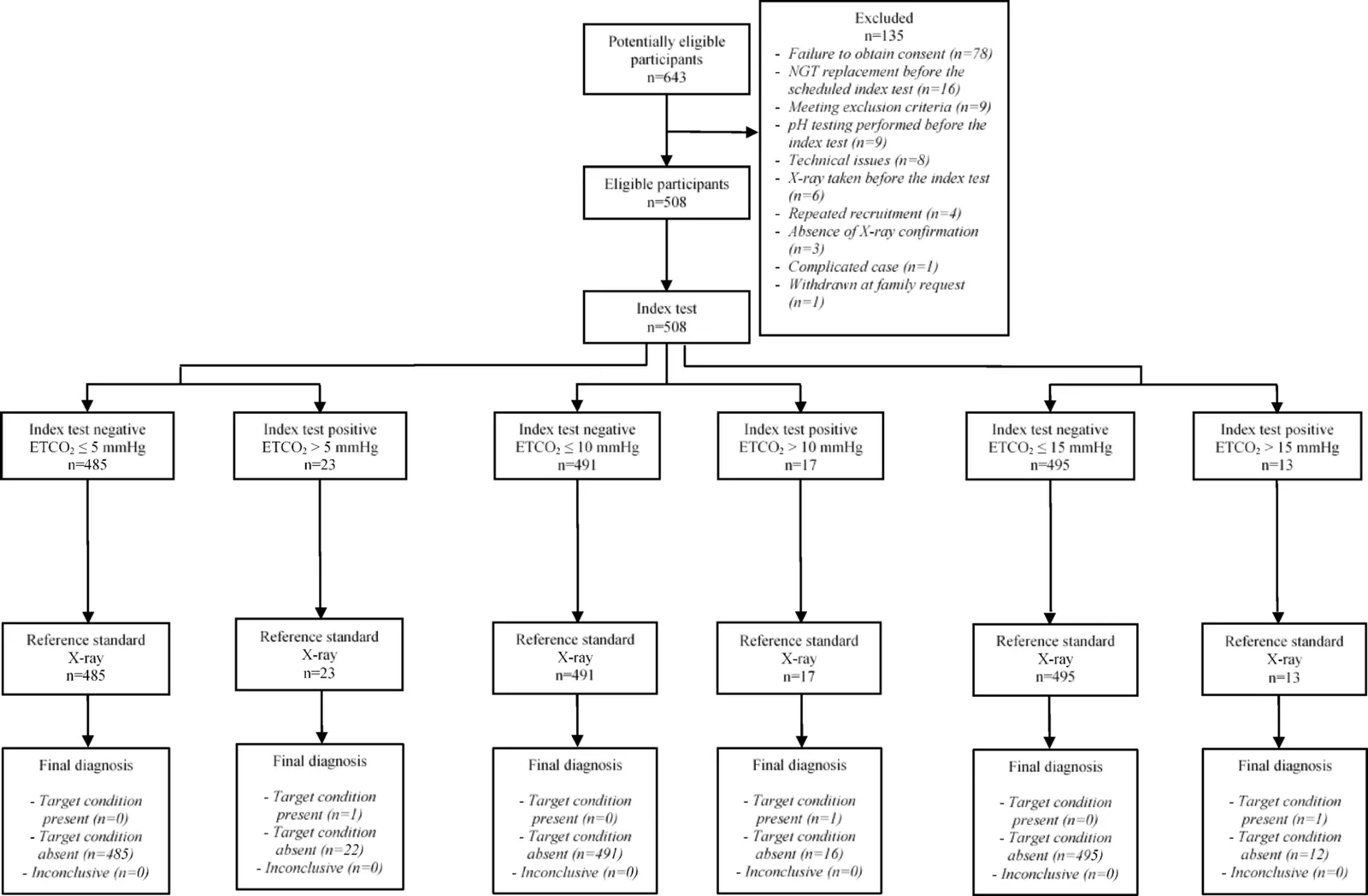
STARD flow diagram

In total, 508 participants were recruited from 39 study sites across 21 public hospitals, including medical or geriatric wards (23.6%), ICUs (33.5%), and EDs (42.9%) (Fig. 2).

Participants ranged in age from 18 to 107 years (mean = 74.9, SD = 15.5) (Table 1). Illness severity, functional status, and cognitive condition were assessed using the Clinical Frailty Scale (CFS) [21], with approximately three-quarters of participants (72.6%) categorised as frail (CFS scores 5–9). For the aspirate pH test, 241 participants (47.4%) had no obtainable fluid aspirate. Among those with aspirate samples (*n* = 267), 206 (77.2%) had a pH ≤ 5.5, while 61 (22.8%) had a pH > 5.5.

Most participants (*n* = 485; 95.5%) recorded an ETCO₂ value ≤ 5 mmHg. Small proportions showed ETCO₂ values within the ranges > 5–10 mmHg (*n* = 6; 1.2%), > 10–15 mmHg (*n* = 4; 0.8%), and > 15 mmHg (*n* = 13; 2.6%). Radiography confirmed correct gastric placement of the NGT in 452 cases (89.0%). One case (0.2%) demonstrated airway placement. A total of 40 cases (7.9%) showed NGT misplacement in the oesophagus, proximal stomach, or small bowel; an additional 15 cases (2.9%) involved a coiled NGT.

### Diagnostic performance of the index tests

**Fig. 3:**
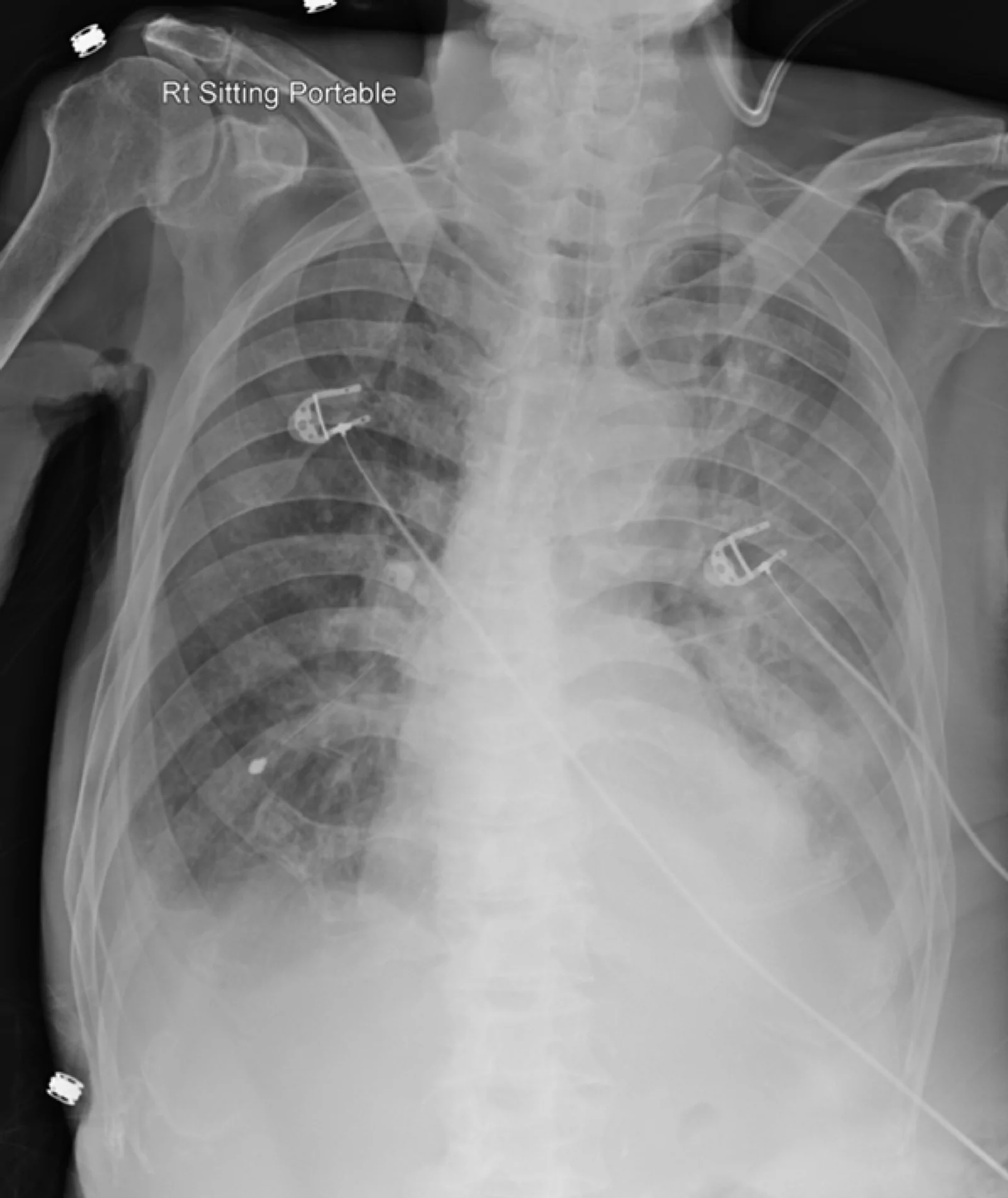
Radiographic evidence of nasogastric tube misplacement, showing the tip of the nasogastric tube projected over the lower zone of the right lung

**Fig. 4:**
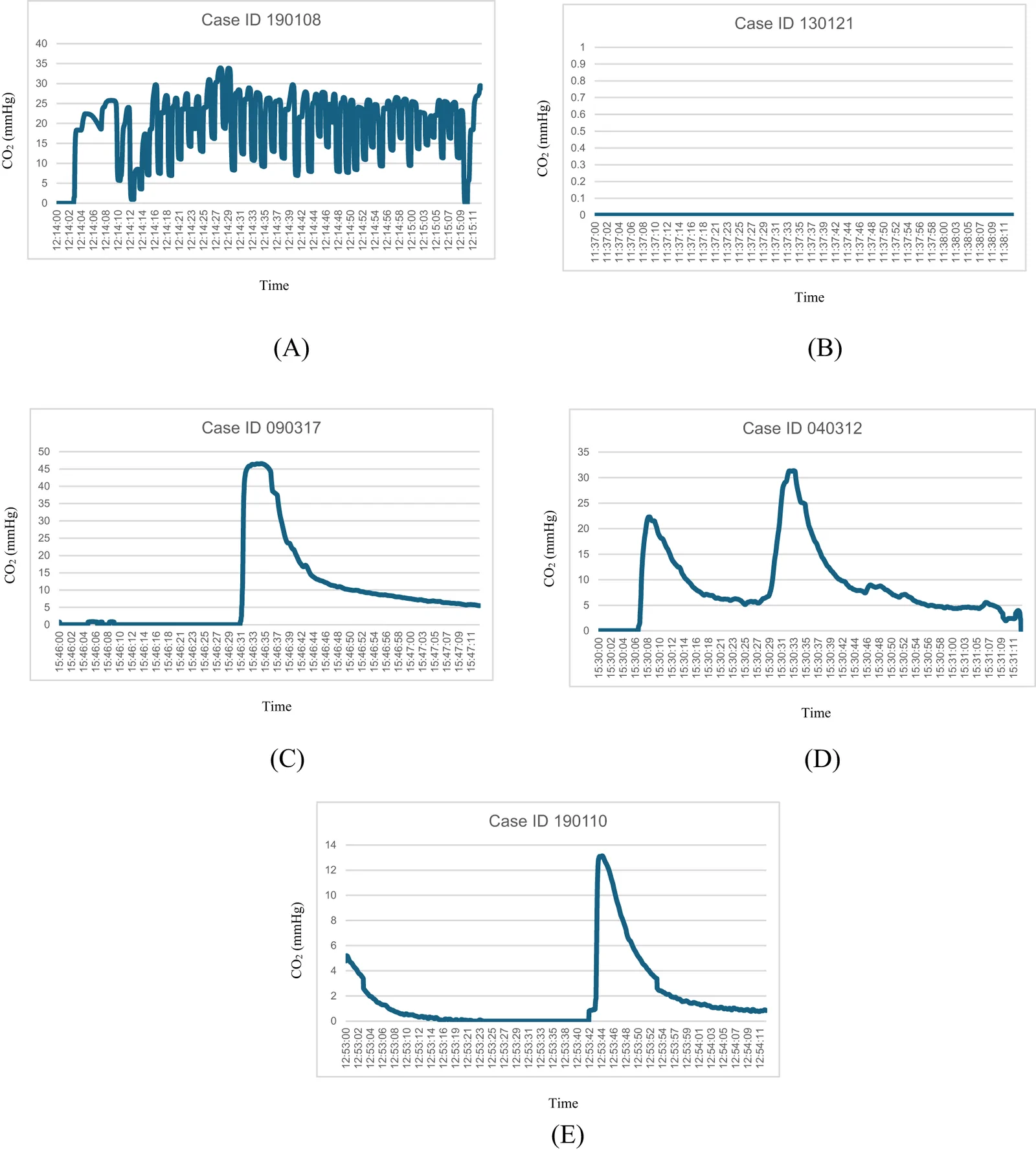
**A**: Capnogram of a true positive case (airway placement; ETCO2 = 32 mmHg). **B**: Capnogram of a true negative case (stomach placement; ETCO2 = 0 mmHg). **C**: Capnogram of a false positive case 1 (stomach placement, ETCO2 = 47 mmHg). **D**: Capnogram of a false positive case 2 (small bowel placement, ETCO2 = 32 mmHg). **E**: Capnogram of a false positive case 3 (nasogastric tube tip above the diaphragm, ETCO2 = 13 mmHg)

Among all study participants (*N* = 508), only one participant (0.2%) was identified with inadvertent airway placement of the NGT, registering an ETCO₂ value of 32 mmHg. Figure 3 presents the corresponding chest X-ray, and Fig. 4A displays the associated capnogram. This case was accurately detected by capnography across all three predefined thresholds (ETCO₂ >5 mmHg, > 10 mmHg, and > 15 mmHg).

No false-negative results were observed for any of the three index test thresholds, yielding 100% in both sensitivity and NPV across all tests (Table 2). The false positive and true negative rates of the three thresholds of ETCO_2_ >5, >10 and > 15 mmHg decreased from around 4% to 2% and increased from around 96% to 97% with a specificity of 96%, 97% and 98%, respectively. Given the nature of low prevalence of inadvertent airway placement of NGT, the PPV of all the index test thresholds were low as well, ranged from 4% to 8%. Nevertheless, the high positive likelihood ratio (ranged from 23 to 42) and the zero negative likelihood ratios of the index test thresholds provide desirable evidence of their diagnostic ability for identifying inadvertent airway placement of NGT.

**Table 2:** Diagnostic performance of using capnography for identifying inadvertent airway placement of nasogastric tube

Diagnostic performance	Diagnostic test thresholds		
	ETCO_2_ > 5mmHg	ETCO_2_ > 10 mmHg	ETCO_2_ > 15mmHg
True positive	1 (0.2%)	1 (0.2%)	1 (0.2%)
False positive	22 (4.3%)	16 (3.1%)	12 (2.4%)
False negative	0 (0.0%)	0 (0.0%)	0 (0.0%)
True negative	485 (95.5%)	491 (96.7%)	495 (97.4%)
Sensitivity (95% CI)	100.0% (2.5% to 100.0%)	100.0% (2.5% to 100.0%)	100.0% (2.5% to 100.0%)
Specificity (95% CI)	95.7% (93.5% to 97.3%)	96.8% (94.9% to 98.2%)	97.6% (95.9% to 98.8%)
Positive predicted value (95% CI)	4.4% (2.9% to 6.4%)	5.9% (3.7% to 9.2%)	7.7% (4.6% to 12.7%)
Negative predicted value (95% CI)	100.0% (99.2% to 100.0%)	100.0% (99.3% to 100.0%)	100.0% (99.3% to 100.0%)
Positive likelihood ratio (95% CI)	23.1 (15.3 to 34.7)	31.7 (19.6 to 51.3)	42.3 (24.2 to 73.9)
Negative likelihood ratio (95% CI)	0	0	0

CI: confidence interval; ETCO_2_, end-tidal carbon dioxide

### Cost evaluation

A cost analysis was conducted to evaluate the total costs incurred to the participants when using radiography only and using capnography together with radiography based on the three ETCO₂ thresholds (> 5, > 10, and > 15 mmHg) for identifying inadvertent airway placement of NGT. The average cost per participant for using radiography only was HK$405.9 ± 168.9, while for the above three thresholds were HK$195.6 ± 85.3, HK$191.3 ± 76.2 and HK$188.3 ± 68.7, respectively (Table 3). The average cost would be saved per patient compared with using radiography only were HK$210.3 (95% CI: 195.0 to 226.2), HK$214.5 (95% CI: 199.4 to 230.0) and HK$217.5 (95% CI: 203.1 to 232.6), respectively (Table 3).

**Table 3:** Hypothetical cost comparison of alternative nasogastric tube placement verification strategies using different ETCO_2_ thresholds for identifying inadvertent airway placement

Approaches	Cost (HK$) per patient	Cost saved compared with using radiography only
	Mean (SD)	Mean (95% CI*)
(1) Radiography only	405.9 (168.9)	
(2) Capnography + radiography (when ETCO_2_ > 5mmHg)	195.6 (85.3)	210.3 (195.0 to 226.2)
(3) Capnography + radiography (when ETCO_2_ > 10 mmHg)	191.3 (76.2)	214.5 (199.4 to 230.0)
(4) Capnography + radiography (when ETCO_2_ > 15 mmHg)	188.3 (68.7)	217.5 (203.1 to 232.6)

CI, confidence interval; ETCO_2_, end-tidal carbon dioxide; HK$, Hong Kong dollars; SD, standard deviation

*95% CIs were estimated using a bias-corrected bootstrapping method

The cost data reported were based on the actual costs involved in radiography and capnography of the study. For approach (1), only radiography costs of the patients were included in the analysis. For approaches (2), (3) and (4), if ETCO_2_ values of the patients were not greater than the thresholds (5, 10 and 15 mmHg, respectively), only their capnography costs were included, otherwise, both capnography and radiography costs were included. Column two of Table 3 shows the average hypothetical cost per patient associated with the four approaches. Column three of the table presents the average costs saved of approaches (2), (3) and (4) compared with approach (1) together with 95% confidence intervals

### Average waiting time for X-ray confirmation of NGT location

The average waiting time from completion of the index test to radiographic confirmation of NGT location was 148.6 ± 150.2 min.

### Adverse events

No adverse events associated with performing either the index test or the reference standard were reported.

## Discussion

While radiography is considered the gold standard for confirming NGT placement, it is not always readily accessible, particularly in convalescent rehabilitation units and community care settings. In emergency care settings, delays in obtaining radiographs and transporting patients to and from hospitals or residential care facilities can delay the initiation of feeding and place additional strain on healthcare resources. Although pH testing of fluid aspirates is recommended as the first-line bedside method for verifying NGT placement, its usefulness is limited when an aspirate cannot be obtained. In this study, 47.4% of tubes yielded no aspirate, and among those with aspirate samples, 22.8% (61/267) had a pH > 5.5 (indicating the NGT may not be in the stomach). These findings are consistent with prior research, including Glen et al., who reported that 19% of pH tests were unsuccessful (either no NGT aspirate or falsely elevated pH level > 5.5) among 1,381 assessments involving 136 patients across intensive care, neurology, respiratory, oncology, and general hospital settings [22].

Our findings corroborate previous research, which the index test demonstrates a satisfactory level of agreement with the radiographic reference standard [23]. A total of 4.3% (22/508) of cases produced false-positive ETCO₂ (> 5 mmHg). Analysis of the associated capnography waveforms indicated that, although a small amount of CO₂ was detected initially, a normal capnographic tracing, characterised by a square-shaped waveform that oscillates at the patient’s respiratory rate [24], was absent on subsequent breaths (Fig. 4C-E). Instead, the waveforms displayed a decrementing pattern with non-square, flat, or near-flat contours, consistent with non-pulmonary placement. Transient CO₂ detection may be attributable to small volumes of residual gas within the GI tract or other recognised causes of false-positive CO₂ readings, such as recent consumption of carbonated beverages or antacids [25, 26].

The sensitivity of the index test in this study was 100%, indicating that all cases of airway misplacement were correctly identified. However, among all participants, only one case (0.2%) was identified with inadvertent airway placement of the NGT. In contrast, a retrospective analysis of radiographs reported a 2.2% incidence of airway misplacement (11/512) [27]. Similarly, a meta-analysis of five trials conducted in acute care settings identified a 1.9% overall rate of small-bore nasoenteral tube malposition in the tracheobronchial tree (187/9,931 placements), with at least five cases resulting in patient deaths. In that review, nasoenteral tubes were defined as small-bore tubes with diameters ≤ 12 F [28]. In the present study, the NGT sizes used were 8 F (0.2%), 12 F (18.3%), 14 F (68.5%), and 16 F (13%), which are commonly used in adult wards, ICUs, and emergency care settings.

In this study, the three diagnostic test thresholds—ETCO₂ >5 mmHg, > 10 mmHg, and > 15 mmHg—achieved specificities of 95.7%, 96.8%, and 97.6%, respectively, indicating that they infrequently produce false indications of airway placement. Adjustment of the ETCO₂ diagnostic threshold from 15 mmHg to 10 mmHg or 5 mmHg did not substantially reduce test specificity. Clinically, applying a lower cutoff would likely result in a greater number of patients with any detectable ETCO₂ being referred for radiographic confirmation—a strategy that may be considered safer and more conservative.

In the current study, aside from the single case of inadvertent airway placement, 40 cases demonstrated NGT misplacement in the oesophagus, proximal stomach, or small bowel; an additional 15 tubes were coiled. NGT malposition is not uncommon [29]. Excessive advancement may result in gastric coiling, retrograde migration into the oesophagus, or passage into the duodenum, whereas insufficient insertion depth increases the likelihood of oesophageal placement, esophageal overfilling, regurgitation, and associated aspiration risk [30, 31]. Taylor and Manara reported that 1,934 radiographs were taken to confirm NGT position in 891 patients, with 15.9% of tubes requiring further advancement (76.9% located in the stomach, 13.3% at the gastro-oesophageal junction, 0.3% in a hiatus hernia, 8.4% in the oesophagus, 0.3% in the pharynx, and 0.6% in indeterminate positions) [32]. These findings underscore the importance of correct insertion length to minimise misplacement.

Compared with radiography, the use of capnography as one of the initial screening tools, followed by radiography only for patients whose ETCO₂ values exceeded the predefined threshold, resulted in substantial cost savings. It should be noted that the cost analysis in this study was designed as a hypothetical comparison of two verification strategies (radiology-only strategy vs. a combined capnography and radiology strategy), in which X-ray would only be performed when the ETCO_2_ value exceeded a predefined threshold and further confirmation was required. As a result, the combined strategy was associated with lower overall costs than the radiology-only strategy because fewer radiographs would be required.

These findings corroborate earlier research demonstrating cost reductions when colorimetric CO₂ detectors are used alongside pH testing compared with radiography alone [12]. Cost analysis specifically for capnography had not been reported in prior research, possibly due to earlier limitations in device availability; however, bedside capnography has become increasingly accessible in recent years. Of note, this study did not include a cost-effectiveness analysis linking costs to patient outcomes. Nevertheless, given the absence of reported adverse events and its ease of use and interpretation, capnography appears to be a safe adjunct to pH testing for excluding airway placement of the NGT.

This study has several strengths. It was conducted prospectively, allowing a more accurate reflection of the true incidence of inadvertent airway placement in the clinical population. Conducted across 21 public hospitals, the multicentre design ensured evaluation of the diagnostic accuracy test across a broad and representative spectrum of patients, enhancing the generalisability of the findings. The sample size was relatively large compared with previous studies assessing ETCO₂ detection for airway placement, thereby increasing statistical precision. Furthermore, data were collected from different patients rather than repeated measurements within individuals, ensuring independence of observations. The use of both continuous waveform capnography (with real-time display) and ETCO₂ values to detect sustained exhaled CO₂ further enhanced diagnostic reliability and supported more accurate clinical decision-making. Verification bias was minimised by applying the reference standard to all participants irrespective of index test results. Additionally, all tests were interpreted independently; ward nurses performing the pH testing were unaware of the capnography findings, and radiograph interpreters were likewise blinded, thereby reducing the risk of bias and strengthening internal validity.

This study has several limitations. First, only one target condition—airway placement—was evaluated in this study, and only one case with the condition was identified. Despite this inherent limitation, the study’s two-year duration and broad institutional coverage enhance the robustness of the findings. Furthermore, while a positive capnography result can help rule out airway placement, it does not confirm correct NGT positioning. Another limitation relates to the variable time interval between the index test and the reference standard, during which NGT position may have shifted due to patient movement or vigorous activities such as coughing or vomiting, potentially leading to oesophageal displacement. Nonetheless, measures such as secure taping of the NGT were implemented to reduce the risk of tube migration (Fig. 1B). We excluded one atypical case involving a patient with a cervical spine injury who had undergone surgery and experienced difficult NGT insertion. Excluding clinically complicated cases may limit the generalisability of findings to the broader population. Given the increased risk of NGT misplacement associated with anatomical abnormalities, we recommend radiographic confirmation of NGT placement in all similar instances. Furthermore, although ETCO_2_ detection demonstrated promising utility in differentiating between airway and GI placement of NGT, its implementation in routine clinical practice may be constrained by the availability of dedicated capnography equipment. As these resources are not universally available across all hospital and community care settings, the wider adoption of this approach may be limited by local resource availability and financial considerations.

Future research is needed to investigate methods to enhance the safety of NGT insertion. Studies should investigate safe and effective alternative methods, including real-time ultrasound-guided insertion, point-of-care ultrasound for confirming NGT position, and video-assisted systems, all of which may improve visualisation and procedural accuracy. Further investigation is warranted to review and refine recommended insertion depths to ensure that current guidelines reflect patient anatomy and clinical contexts. Additionally, comparative studies examining mainstream versus sidestream capnography could yield valuable insights into their relative accuracy and clinical utility in detecting inadvertent airway placement.

The diagnostic performance of capnography supports its incorporation into routine practice immediately after NGT insertion, both in hospital and community care settings. Findings from this study highlight the potential system-level impact of adopting capnography: among 135 care-home residents admitted to the EDs, 85 (63%) were transferred specifically due to issues related to NGT dislodgement or uncertainty about placement, primarily because aspirates could not be obtained for pH testing. Implementing an additional verification method such as capnography could substantially reduce avoidable ED visits, decrease reliance on emergency imaging, and minimise the physical and psychological distress associated with hospital transfers for vulnerable residents.

## Conclusions

This study demonstrated that capnography has high sensitivity and specificity for distinguishing airway from GI placement of NGTs across critically ill patients, general-ward inpatients, and individuals admitted to EDs. Capnography may facilitate earlier identification of airway placement, thereby supporting timely tube repositioning or reinsertion. In addition, the findings suggest that its use may be associated with cost savings. Given its diagnostic performance and ease of use, capnography may be considered a first-line tool for excluding inadvertent airway placement of NGTs when used alongside pH testing of aspirates.

## Supplementary Information

Below is the link to the electronic supplementary material.

Supplementary Material 1.

## Data Availability

The datasets used and/or analysed during the current study are available from the corresponding author on reasonable request.
